# Physiological and Behavioral Synchrony Predict Group Cohesion and Performance

**DOI:** 10.1038/s41598-020-65670-1

**Published:** 2020-05-21

**Authors:** Ilanit Gordon, Avi Gilboa, Shai Cohen, Nir Milstein, Nir Haimovich, Shay Pinhasi, Shahar Siegman

**Affiliations:** 1 Department of Psychology, Bar-Ilan University, Ramat-Gan, Israel; 2 The Gonda Multidisciplinary Brain Research Center, Bar Ilan University, Ramat-Gan, Israel; 3 The Music Department, Bar Ilan University, Ramat-Gan, Israel; 4 The Psychology Department, Rupin College, Emeq-Hefer, Israel; 5 The Department of Computer Science, Bar Ilan University, Ramat-Gan, Israel

**Keywords:** Social behaviour, Cooperation, Human behaviour

## Abstract

Interpersonal synchrony contributes to social functioning in dyads, but it remains unknown how synchrony shapes group experiences and performance. To this end, we designed a novel group drumming task in which participants matched their drumming to either predictable or unpredictable tempos. Fifty-one three-person groups were randomly assigned to one of two conditions: synchronized or asynchronized drumming. Outcome measures included electrocardiograms and self-reports of group cohesion and synchrony. The drumming task elicited an increase in physiological synchrony between group members (specifically their hearts’ interbeat intervals). We also found that physiological synchronization and behavioral synchronization predicted individuals’ experience of group cohesion. Physiological synchrony also predicted performance in a subsequent group task that involved freely drumming together. The findings suggest that the behavioral and physiological consequences of synchronization contribute to the formation of group bonds and coordination. They also confirm that insights from translational social neuroscience can inform our knowledge of the development of cohesive and efficacious groups.

## Introduction

Interpersonal synchrony is widespread across human culture and nonhuman groups. It is an evolutionary-based mechanism that facilitates social bonding, cohesion and exchange (i.e., it is a “social glue”)^1–4^. Interpersonal synchrony first emerges during infancy via parent-child synchrony and later becomes a cornerstone of social development. This early experience of synchrony affects brain development and becomes crucial for developing self-regulation, empathy and symbolic skills^5,6^. Interpersonal synchrony has a profound social impact later in life (e.g., in adolescence and adulthood), leading to increased pro-sociality and social cognition^7–9^.

Throughout the lifespan, synchrony is largely facilitated within group settings. Group processes, including cooperation and coordination, are central components of societal development and flourishing. Humans enter into group dynamics and processes from birth, and these processes become embedded into the human brain as blueprints of social interaction^10^. However, despite the known implications of group processes for an individual’s social life, there is little research on how psychophysiological synchrony in group settings shapes group processes during real-time shared activities.

Social grouping is universal across cultures^10,11^ with evolutionary advantages and implications for human biology^11–13^. Groups that are more cohesive tend to be more effective than groups that are less cohesive^14^. The topic of group cohesion has a longstanding presence in the literature, with volumes of theory and research on living units, task groups, group therapy and group dynamics^15–19^. For example, there is robust evidence that cohesion contributes to performance, productivity, and behavioral change^15,20–23^. In essence, a group’s level of cohesion provides a window into the quality and quantity of the social bonds within the group^17,24^. Thus, here, we suggest that just as we inform theories and intervention regarding pair bonding with physiological sciences^25–27^, we should also introduce a biobehavioral perspective to the group cohesion literature. Toward that end, our study builds on prior research on interpersonal synchrony by integrating it with group cohesion research. Specifically, considering that group work and cooperation are crucial for everyday life, identity and action, we aim to examine behavioral and physiological synchrony with reference to group cohesion and performance theories.

On a neural level, the adaptive prosocial benefits of synchrony are linked to the activation of reward signals in the brain and mirror neuron activity—both of which may promote future adaptive social interactions and bonding^28–30^. Furthermore, neural components that comprise the “social brain”^31^ are active during synchronous motor activity, including the increased midline activation of structures such as the ventromedial prefrontal cortex, hippocampus, supplementary motor area, primary somatosensory cortex (extending into the primary motor cortex), posterior cingulate and precuneus^32^. An electroencephalogram (EEG) study found synchronous oscillatory brain activity in the theta and delta bands as guitar players performed duets^33^. Oscillatory brain activity in the alpha and mu bands has been associated with effective social coordination^34,35^. Brain-to-brain synchronization at the dyadic level has been shown to support group-specific dynamics, including leader emergence^36^ and student engagement in class^37^. Beyond neural activation, coupling in peripheral measures of the Autonomic Nervous System (ANS), such as cardiac and respiratory patterns, provides physiological correlates of interpersonal action coordination. For example, when people sing in unison, their heartbeats accelerate and decelerate simultaneously^38^.

Synchronized behavior between individuals in a dyadic joint activity increases cooperation, liking and rapport^39,40^. In one study, coordination during a finger-tapping task predicted affiliation ratings, prosocial behavior and cooperation^29^. Finger-tapping tasks are a relatively common way to explore sensorimotor synchronization^41,42^. However, this method is limited for two prime reasons. First, the affect evoked from participants is quite neutral, and second, the method is usually performed using an experimenter or metronome. Therefore, these paradigms lack the naturalistic and ecologically valid components that are a part of real-life group interactions.

To overcome these limitations, it has been suggested that joint music making may constitute a promising experimental platform for implementing ecological and fully interactive scenarios that capture the richness and complexity of human social interaction^43^. Shared musical experiences are a form of social collaborative behavior that compels individuals to anticipate and adapt to each other’s behavior. The perceived synchrony during a shared musical paradigm, especially when there is a shared goal to produce synchrony, may provide immediate feedback to group members indicating successful cooperation and therefore reinforcing the group’s cooperative behaviors^44^. From the perspective of music theory and research, when people make music together in small ensembles, these ensembles act much like teams or work groups^45,46^ in which there is continuous attention and sensitivity between the performers in the ensemble. This phenomenon has been described as an attempt to align one’s emotional states with those of the coperformers, with the end goal of achieving musical cohesion^47^. As a result, shared musical experiences constitute a fitting paradigm in which synchronization between group members can be considered critical for successful social interaction, resulting in group cohesion^39^.

As we previously noted, little is known about how physiological synchrony manifests in a real-time interactive group setting because most studies have assessed rhythmic synchronization only in individuals or dyads^10^. Even more scarce are studies regarding the links between behavioral and physiological synchrony among group members and the group’s resulting cohesion. A 2017 review on ANS synchrony^48^ noted that only two out of 61 studies focused on ANS synchrony in groups instead of individuals. The following are proof of concept studies that have pointed to ANS synchrony as a potential marker of group outcomes. In a study of a single group comprising four students, heart rate variability (HRV) was monitored during 18 meetings over 6 months, and HRV synchrony was found to be related to lower ratings of teamwork and productivity^49^. Another study of 10 four-person teams who performed a military training task showed that better team performance was associated with higher physiological synchrony in measures of respiratory sinus arrhythmia^50^. Recently, a study in which 51 triads were instructed to build origami boats together found that members of a newly formed team exhibited synchronized physiological measures during the task. Furthermore, synchrony of skin conductance was associated with group tension and negative affect, whereas synchrony of facial muscle activity (smiling) was positively associated with group cohesion^51^. In a separate study, dyadic-level ANS synchrony has been shown to support larger group-level phenomena, including mass participation in cultural rituals^52^. Thus, examining ANS function continuously in dyads among groups during naturalistic interactive states can advance our understanding of the biological and behavioral processes that are unique to the group level.

In the present study, our overarching goal was to deepen our fundamental understanding of how multimodal synchrony contributes to group bonding by assessing how behavioral and physiological synchrony enhance group cohesion and performance. By doing so, we aim to bridge the gap in the literature between the outcomes of behavioral and physiological synchrony and the processes that are unique to groups^53^. Toward that end, we used a social neuroscience perspective and utilized a novel musical paradigm to investigate interacting groups. We had three specific aims. First, we aimed to measure how group cohesion and performance change as a result of manipulating the level of behavioral synchronized drumming in groups. Second, we aimed to assess whether physiological synchrony of interbeat intervals (IBIs) of the heart – a cardiac measure of the ANS derived from electrocardiograms (ECGs) – emerged between group members during the drumming task and to measure whether this physiological synchrony had effects beyond behavioral synchronization and contributed to group cohesion and performance. Third, we aimed to test whether IBI and behavioral synchrony during the drumming task enhanced an objective measure of coordinated drumming in a free improvisation activity that followed the study’s task.

**Figure 1:**
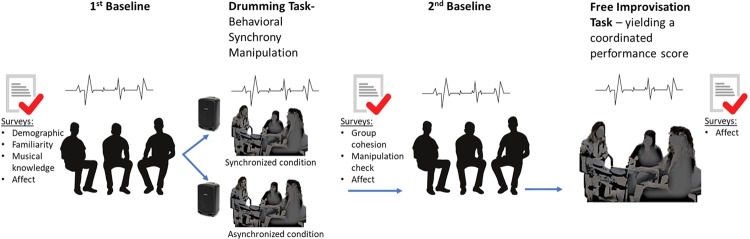
An outline of the study’s procedure. Before the music task, we assessed each group member’s demographic information, affect, prior familiarity with each other, and prior musical knowledge in self-reported questionnaires. Next, we recorded each participant’s ECG during a 5-minute baseline in which they were instructed to sit together and relax. The ECGs were monitored continuously from the first baseline until the end of the study. Following the 1^st^ baseline measurement, the drumming task began and lasted 4 minutes. After the drumming task, the participants completed manipulation check questions that assessed perceived drumming synchrony, the main outcome measure of group cohesion, and affect surveys. Next, a second 5-minute baseline period that was identical to the first one began. Then, the participants were instructed to freely play together on the drums for 4 minutes, which allowed us to derive an objective group performance measure of coordination in a new improvisation task. Affect was then reported via self-reports for a final time in the study. Figure credits: [“Yuii]/Shutterstock.com”; “[flatvector]/Shutterstock.com”; “[NadzeyaShanchuk]/Shutterstock.com”.

To test these aims, we conducted a drumming task with 51 three-participant groups in which IBI data were continuously collected from all participants throughout the study. We asked participants to match their drumming (on their individual drumming pads within an electronic drum set shared by the group) to a tempo that was presented to the group through speakers. For half of the groups, the tempo was predictable (i.e., beats at a steady tempo), and thus, the resulting drumming and its output were intended to be synchronous. For the other half, the tempo was unpredictable (i.e., beats at a constantly changing tempo that were practically impossible to follow), so the resulting drumming and musical output were intended to be asynchronous. This drumming task enabled us to manipulate the level of behavioral musical synchrony in the group and assess the dynamics of changes in IBI for each participant throughout the experiment. Following this shared musical task, we asked each participant to rate his or her perceived level of group cohesion. In line with previous research, we hypothesized that synchronous drumming (task condition) and group IBI synchrony would enhance group cohesion. Since prior theory and research show that physiological synchrony can emerge spontaneously during group interactions, we expected IBI synchrony in the groups to be elevated during the drumming task compared to the baseline. See Fig. 1 for a visual depiction of the study’s procedure.

## Results

Table 1 presents descriptive statistics for the main outcome measures in our model across the two task conditions. It includes results from independent sample *t*-tests that compared mean scores across task conditions. See Tables S1 and S2 in the supplementary materials for individual-level and group-level correlations of the main outcome variables.

**Table 1:** Group Cohesion, IBI Synchrony, Perceived Drumming Synchrony and Drumming Together across Synchronized and Asynchronized Task Conditions.

	Synchronized		Asynchronized		*t*(df), sig.
	*M*	*SD*	*M*	*SD*	
Group IBI Synchrony	0.17	0.15	0.16	0.15	0.18(45), *p* = 0.85
Group Cohesion	4.26	0.98	3.85	1.08	2.4(147), *p* = 0.016
*Perceived Synchrony	2.75	0.84	2.45	0.77	2.19(147), *p* = 0.03
*Drumming Together	439.6	316.94	318.3	195.33	2.736(142), *p* = 0.007

*Note*. * “Perceived synchrony in drumming” and “drumming together” were manipulation validation measures for the drumming task conditions.

### Predicting group cohesion from IBI synchrony in the drumming task condition

We used a linear mixed model analysis in IBM’s SPSS25 to predict group cohesion (Level 1) from our task condition (synchronized versus asynchronized drumming) (Level 2) and Group IBI synchrony (Level 2). This model accounts for the fact that the data are nested (i.e., individuals within specific groups). A scaled identity matrix was selected as the covariance structure chosen for this mixed model. Level 1 predictors were centered on the group mean. We defined independent predictors as fixed effects because of the small number of participants in each group (i.e., 3). To control for potential confounding effects at Level 1, we added prior familiarity and musical knowledge as independent predictors. The results are given in Table 2. The model results were significant with and without the controls.

**Table 2:** Predicting Group Cohesion from Behavioral and Physiological Synchrony.

	Estimate	*SE*	*t*(df)	sig.	95% CI
Task Condition (0 = sync; 1=async)	−0.46	0.22	−2.07(43.35)	0.044	−0.9– −0.01
Group IBI Synchrony	1.76	0.73	2.41(43.78)	0.020	0.29–3.2
Prior Familiarity	0.24	0.15	1.56(88.5)	0.121	−0.06–0.55
Musical Knowledge	−0.14	0.09	−1.6(89.2)	0.113	−0.32–0.03

### IBI synchrony during the drumming task

**Figure 2:**
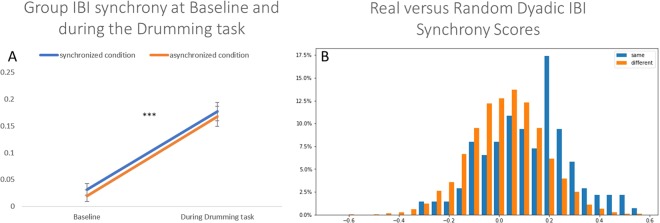
(**A**,**B**). In panel A, we present changes in the group-level physiological synchrony (y-axis) from the baseline (in which group members were asked to sit together and relax for 5 minutes) to the drumming task. There was a significant increase in IBI synchrony from the baseline in all groups during the drumming task, which was unrelated to the drumming condition (*t*(47) = −5.37, *p* = 0.0001, 95% *CI* = −0.2–0.09.). In panel B, we present a frequency distribution showing the percentage of dyads (y-axis) that had any given IBI correlation score (x-axis). In blue, we show the real interacting dyads from the same group, and in orange, we show all possible randomly created dyads from different groups. These two samples are significantly different from each other with higher-level synchrony scores in real dyads. In combination, both panels show that IBI physiological synchrony in the drumming task was higher than baseline synchrony in the same groups and was not spurious due to similar task conditions across groups. ***p < 0.0001.

In Fig. 2A, we demonstrate that in both conditions, there was a significant increase in group IBI synchrony from the baseline, above and beyond the study’s manipulation. Group IBI synchrony was not related to the drumming condition. Due to this nonsignificant relationship, a mediation model in which the study’s conditions lead to IBI coordination in the group, which in turn influences group cohesion, is precluded.

To test for the possibility that group correlations in IBI were due to “real” aspects of the groups’ dynamics and were not spurious (e.g., due to group members experiencing a similar group state across all groups), we compared all dyadic correlations among real group members to a set of all the possible noninteracting random dyads (i.e., not derived from real groups). Toward that end, we computed a random dataset of 9,732 dyadic correlations (see Fig. 2B). We then compared the real dyadic correlations to randomly calculated correlations using the Kolmogorov-Smirnov test and checked against the null hypothesis that the data come from the same distributions, which yielded a significant difference between random and nonrandom dyads: *Sig*. = 4.3*10^−10^. As seen in the frequency graph in Fig. 2B, the average correlation of nonrandom dyads was *M* = 0.142 (*SD* = 0.01) compared to random dyads *M* = 0.0498 (*SD* = 0.002). This result indicates that the correlation coefficients achieved in real dyads are indicative of the specific interactive dynamics within each group and not to spurious effects, including the effect of being in the same room, engaging in similar tasks, hearing a similar beat, or drumming across all groups.

**Figure 3:**
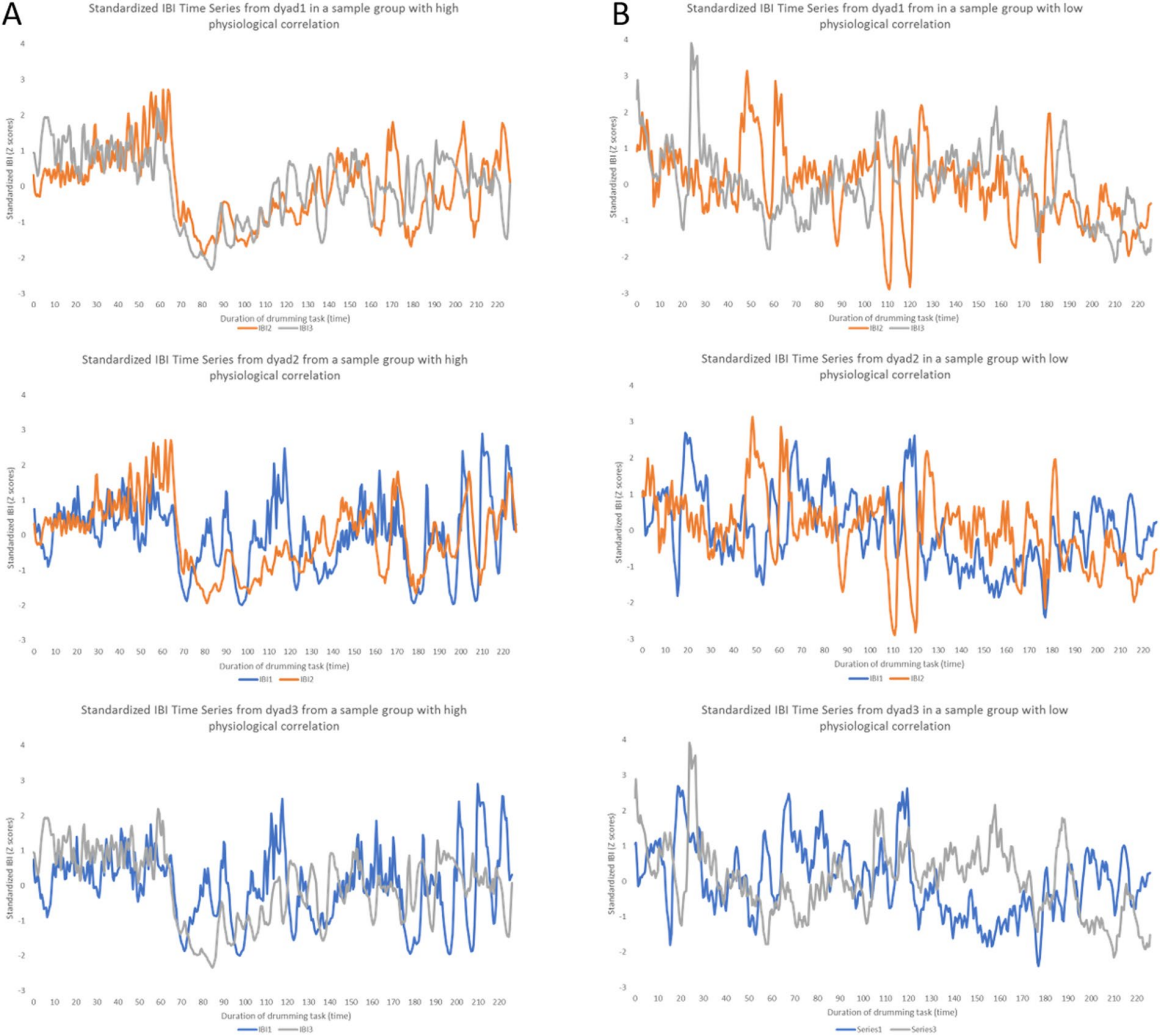
In panels (A,B), we present the time series of the interbeat intervals (IBIs) of the heart from all dyads within the sample groups participating in the drumming task. Each group includes three dyads, which are presented separately. We present the IBI time series for every dyad in the group so that the level of dyadic correlations comprising the groups’ average can be viewed. In panel A, we present dyads from a group with a high level of physiological synchrony. In panel B, we present dyads from a group with a low level of physiological synchrony. The IBI time series was derived from the ECG signal for each group member throughout the duration of the drumming task. The y-axis represents standardized IBI (Z-scores of intervals in seconds), and the X-axis represents the time elapsed from the beginning of the drumming task in seconds. As shown, there is a higher level of convergence and covariation for dyads on the left panels than for dyads on the right panels.

**Figure 4:**
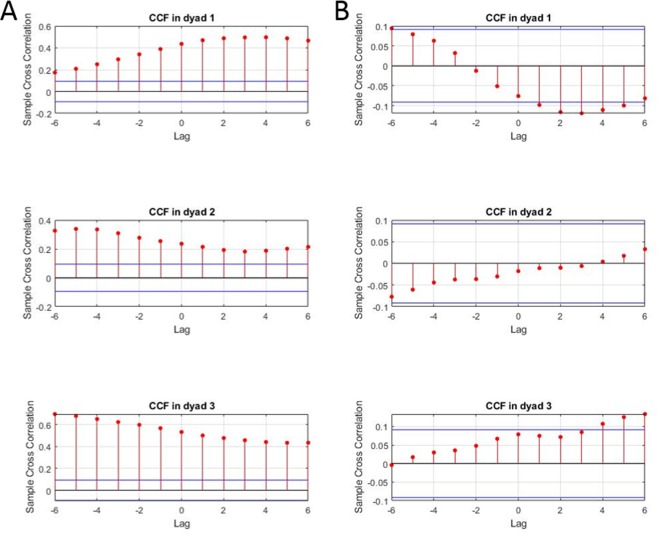
This figures shows the results of cross-correlation function (CCF) analyses performed on the IBI time series of all possible dyads from the two sample groups presented in Fig. 3 during the drumming task. The y-axis is the level of correlation. The x-axis is the temporal lag of 3 seconds (from −6 to +6 data lags) of the CCF. The two horizontal blue lines in each graph represent the 95% confidence interval. Correlations that extend vertically beyond the blue lines are significant. Panel A presents an example of three CCF analyses in dyads from a group with a high level of IBI synchrony. Panel B presents an example of three CCF analyses in dyads from a group in which there was a low level of IBI synchrony. This figure shows that dyads from groups with high IBI synchrony have higher and more significant CCF scores than those from groups with low IBI synchrony.

Figure 3 provides a description of the IBI time series in all dyads comprising two selected groups, and Fig. 4 provides a description of the cross-correlation function (CCF) analysis performed on each of these dyads’ time series.

### Predicting group coordinated performance from IBI synchrony and the drumming task condition

Group performance was assessed during a second group task (and not during the drumming task) in which group members were asked to play freely together without any further structure or tempo to follow (i.e., free improvisation). This task provided us with an independent performance measure indicating group coordination.

#### Coordinated performance measure yielded from electronic MIDI files

Each free improvisation task resulted in a digital MIDI file that was analyzed by deriving a measure of drumming coordination (i.e., the number of times individuals in the groups hit the drums together within a window of 30 milliseconds). We used a hierarchical linear regression model *strictly* at the group level to predict drumming coordination during the improvisation task from two predictors that were measured during the preceding drumming task: IBI synchrony and the task condition. The regression model was significant overall (*F(2,44)* = 3.99, *p* = 0.025), explaining 15.4% of the variance in the predicted variable. Group physiological synchrony during the drumming task was positively related to drumming coordination in the improvisation session (*B* = 942.71, *Beta* = 0.31, *t* = 2.25, *p* = 0.029, *95% CI* = 99.40–1786.01). The previous drumming task condition did not significantly predict drumming coordination in the improvisation session (*B* = 225.39, *Beta* = 0.24, *t* = 1.77, *p* = 0.084, 95% *CI* = −31.26–482.05).

### Validation of the drumming task condition – manipulation checks and controls

#### Manipulation checks

As seen in Table 1, the drumming task condition was associated with the reported level of perceived synchrony among group members such that in the synchronous drumming condition, individuals reported a stronger experience of synchronized drumming than individuals in the asynchronous condition. For an additional, more objective manipulation check, we analyzed the digital MIDI file for each group and extracted the number of times all group members drummed together during the task (details of the extraction procedure are presented in the Methods section). Indeed, group members drummed simultaneously more often in the synchronous condition than in the asynchronous condition (Table 1).

#### Affect

To exclude the possibility that our results represent changes in positive or negative emotions among group members that were specific to either the task condition (perhaps the nonsynchronous drumming condition was frustrating and contributed to an elevation of negative emotions, which may explain our results), we tested whether our task condition was associated with changes in affect. We found that for negative emotions only, there was a significant decrease following the drumming task in both the synchronous and asynchronous task conditions. Positive affect did not change from before the drumming task to after it (see the supplementary results for full details of the repeated measures ANOVA performed). These results indicate that the asynchronous task condition was not associated with an increase in negative emotionality or a decrease in positive emotionality, which may account for our results.

## Discussion

Our results highlight the importance of physiological and behavioral mechanisms of synchronization that support the development of group cohesion and performance. Using an ecologically valid real-life group setting via a novel synchronous vs. asynchronous musical paradigm, we showed that a manipulation in behavioral synchrony and emerging physiological coordination in IBI between group members predicted an enhanced sense of cohesion among group members.

These findings have several important implications. First, they build on the large volume of previous work on interpersonal synchrony from the dyadic level^54,55^ and extend it to the group level. We gained an understanding of how synchrony, in different modalities, is associated with cohesion, resulting in coordinated performance in a complex group system. Second, noting that behavioral synchrony and physiological synchrony are both unique and independent predictors of group cohesion, we now have a more precise understanding of the distinct avenues by which group therapies or interventions can work. Since synchronous drumming is frequently used in group music therapy to enhance feelings of “togetherness” and cohesion^56,57^, such clinical work can benefit from the physiological augmentation of the phenomenon investigated in this study. Third, we offer a novel group musical research paradigm that is nonverbal and that is specifically designed for eliciting different levels of behavioral and perceived synchrony within groups. This paradigm is well suited to explore social bonds at the group level through online physiological functioning; in this way, it can expand our knowledge about group processes from a social neuroscience perspective.

Overall, physiological synchrony in IBI increased in all groups during the drumming task compared to the baseline and was above what could be expected randomly. Prior research suggests that physiological synchrony can emerge as a feature of group cooperative tasks or active joint activities^48^. In dyads, it has been shown that increased heart rate synchronization and facial muscle synchronization occur automatically without direct interaction when individuals merely sit side by side and watch movies together^58,59^. Our drumming task was not a passive task and involved drumming together and face-to-face interaction; therefore, an increase in IBI synchronization may have been the result of the shared state as well as the active participation in a group task, regardless of whether the rhythms were predictable or not. Perhaps what matters for the formation of physiological synchrony is not the actual action of drumming together but rather the intention to do so. The seminal work by Tomasello and colleagues highlighted the human drive to understand and share others’ intentions as the main factor that differentiates human social cognition from animal social cognition^60^. This ability is at the core of humans’ unique evolutionary instinct to cooperate with others^61^. During nonformal forms of music making, which are a fundamental part of the human experience beginning in infancy^62^, interpersonal synchrony arises. As such, these musical forms may have evolved to support social bonding via synchronization^63,64^. Therefore, when we participate in group music making later on in life (e.g., during cultural rituals), we synchronize with others^62,63^, which in turn supports our sense of group cohesion. This intrinsic human motivation to share experiences with others gives rise to synchrony during shared musical states, which has been suggested as a mechanism that allows participating individuals to continuously monitor and share collective intention^63^. Future studies could investigate whether physiological synchrony is also a consequence of shared intentionality during joint music making.

The lack of association between the behavioral and physiological synchrony reported in our study highlights the importance of incorporating physiological responses into behavioral research, as physiological functioning can offer insights into behaviors that are not readily captured by self-reports or observations. The independence of behavioral and physiological synchrony reported here fits with the prior inconsistencies in the literature describing the relationship between behavioral and physiological synchrony and suggests that direct associations between the two should not always be expected^48^. The concept of physiological synchrony is itself a multifaceted structure, and similar to behavioral synchrony^65^, the results may vary according to the type of physiological measure used, the way synchrony is calculated and the context assessed^66^. It is possible that physiological and behavioral synchrony were unrelated in our study due to the specific measures chosen. It is also possible that as synchronization dynamically changes over time^5,51^, the computation of synchrony does not capture potential moments when behavior and physiology align. Conversely, each modality of synchrony can work independently in certain states to uniquely contribute to group cohesion.

The motivation to synchronize and individual differences in the willingness to synchronize were not the targets of the current study, which may have contributed to the increase in IBI synchronization in all groups despite the actual differences in coordinated drumming. A strong motivation to synchronize when drumming together with social partners has been previously observed—a study showed that children as young as 2.5 years old adjusted their drumming tempo and accuracy better when drumming with a social partner than when using a metronome^67^. The authors of the study suggested that drumming together with a social partner elicits a specific human motivation to synchronize during joint rhythmic activity^67^. Behaviorally, individuals in social settings that involve cooperation have been shown to quickly and automatically converge to synchrony – as was the case in a “sheep herding” game in which dyads distinctly shifted from asynchrony to synchrony, which allowed for successful completion of the game^68^. An intrinsic motivational component to synchronize, together with an opportunity for face-to-face interaction with others, can also elicit synchronization^2,69^ and are therefore factors that we can suggest as comprising the potential background in which physiological synchrony may develop^37,70^. Interestingly, in the present study, this motivation may have emerged regardless of the actual rhythm patterns presented. That is, at times, participants tried to synchronize and later anecdotally reported that they played together, even when the rhythm was rather random and sporadic and did not allow steady synchronization to occur. Future studies should shed light on which precise characteristics of shared group interactions and of the individuals engaging in them elicit increased IBI synchrony. It is also equally important to model these states against maladaptive states in which the motivation to synchronize is lacking. Alternatively, when a representation of the joint activity is not formed, physiological synchrony cannot emerge or bring about effective group cohesion. In future studies that employ the research paradigm developed here, it will be possible to manipulate the degree of asynchrony of the rhythmic patterns that emerge to gauge whether there is a point at which the human motivation to synchronize or perceived cohesion begins to deteriorate. Inversely, future research could manipulate the degree of motivation to be in rhythm and assess whether it influences cohesion, regardless of the degree of the tempo’s predictability.

In the present study, physiological synchrony early in the group process predicted increased behavioral coordination in drumming in a subsequent improvisation task. Thus, beyond self-reported attitudes, we provided an additional objective measure of enhanced coordinated performance in groups predicted by physiological synchrony. This result corresponds to previous studies showing a positive association between IBI synchrony and team performance^48^. This result also fits with prior literature showing a strong relationship between group cohesion and performance^14,20^ as well as reports that rhythmic synchrony, in addition to fostering cohesion, can improve skills that allow humans to better cooperate and pursue joint goals^71^.

What are the possible underlying mechanisms of physiological group synchronization in our study? According to polyvagal theory^72^, IBI functioning during nonthreatening social interactions is considered a part of the “social engagement system”, a phylogenetically advanced regulatory capacity that evolved to support social interactions. This system allows for subtle changes in IBI according to the demands of the social interaction. These subtle modulations are in part a result of specific neuromodulators—oxytocin and vasopressin—which are extensively implicated in bonding and sociality^73,74^. Future studies should measure these neuropeptides, their genetic markers, and the brain structures that involve their regulation to obtain a better sense of the biological mechanisms underpinning IBI synchronization. A more recent model^11^ of the herding brain suggests that several neuronal, hormonal, cognitive and behavioral mechanisms support group processes. Some of these mechanisms are partially dyadic by nature, including emotion contagion and behavioral alignment as well as brain-to-brain synchrony, while others are at the individual level, including conformity and brain activations in three key neural networks. Surprisingly, despite the group-level nature of the herding phenomenon, there are few suggestions for the possible biological mechanisms computed at the group level, as this level of representation requires different avenues of analysis of data from what has been done thus far (an exception includes the quantification method suggested here^75^). Behavioral synchronization and performance in the current study were measured at the group level, and physiological synchronization was based on dyadic computations (which is common in interpersonal synchrony research) and then averaged to the group level in order to capture the variance in the group. Future studies may seek to explore and develop quantification methods that can capture group-level biological mechanisms.

Since the main physiological measure in the current study was IBI, which is influenced dynamically by both the sympathetic (S) and parasympathetic (P) branches of the ANS^76^, we were unable to determine how the balance of P and S influences IBI function. We know that the autonomic branches are not regularly reciprocally controlled^76^, especially in complex psychological processes such as synchrony, which may involve either the independent activation or the coactivation of the two ANS modalities^77–80^. To reach a deeper mechanistic understanding of the sympatovagal balance underlying synchronization, future studies should incorporate ANS measures that are considered strictly P or S in nature. Despite the nonspecific nature of IBI, it is an ANS marker that can be derived noninvasively. It is a simple and easily computed time-domain continuous signal that can be utilized in many groups and in various research and clinical settings, which represents a strength of the current research from a translational perspective.

It should be noted that physiological synchrony is not always a predictor of better performance outcomes. In fact, physiological synchrony can be detrimental. For example, in the case of stress contagion, dyadic physiological covariance between mothers and infants shows that a mother’s stressful experiences can be “passed on” to her infant via reciprocal interactions and that this transference is linked to physiology^81^. Similarly, witnessing another person’s stressful experience can induce a “contagious” physiological stress response in the observer^82^. Other physiological markers beyond IBI, such as skin conductance^51^, may shed light on the “dark side” of synchronization at the group level and help pinpoint the contexts in which physiological synchrony can derail group bonds. By the same token, there is a “dark side” of group cohesion that can also serve to facilitate negative attitudes and harmful behaviors toward members of an out-group^83,84^. Future studies are necessary to reach an understanding of how physiological synchronization may have negative and dangerous consequences.

The present study was hindered by several limitations. First, there was a low level of prior familiarity between some group members, and levels of prior familiarity were not equally distributed across task conditions. Although we controlled for prior familiarity in our statistical analyses and found that it did not have a significant effect, future studies that test the initial stages of group formation should aim to test groups that consist only of strangers. Second, our results are primarily relevant for newly formed groups. The study included participants who did not know each other well; therefore, it is unclear whether these effects would be similar in recurring or well-acquainted groups, which should be tested in future research. Therefore, a critical future direction of this work is to test whether our findings can be generalized to other types of groups. Third, we did not control for gender or gender composition in the groups. Future studies should aim to test for gender effects, as there is some evidence that gender composition may influence both synchrony and cohesion^85,86^. A strength of our work is that we demonstrated that physiological synchrony can generalize to coordination in subsequent group tasks such as improvisation. This finding is particularly significant due to the unique and independent contribution of physiological synchrony to performance unrelated to behavioral synchrony. Future intervention models may consider including a physiological “augmentation” component unrelated to induced behavioral synchrony. The results of the present study provide an empirical basis for a more detailed understanding of the physiological and behavioral dimensions of interpersonal synchrony and how they may contribute to group bonding, shape cohesion and influence subsequent performance.

## Method

### Participants

The Bar-Ilan University Department of Psychology Ethics Committee approved this study. All methods were conducted in accordance with the approved ethical guidelines. Each participant provided informed consent. In this study, 153 participants were nested in 51 groups of triads. We dropped one group from the analyses because one participant in the group reported high levels of distress and could not complete the study. Due to technical or biological issues in the physiological data analysis, we could not use physiological data from an additional 3 groups. Therefore, the final analyses were conducted on data from 141 participants nested in 47 groups. Participants were undergraduate students in the Department of Psychology at Bar-Ilan University. The total sample consisted of 32 (23%) males and 119 (77%) females. The average age was 22.6 years old (*SD* = 2.28) for males and 22.4 years old (*SD* = 1.8) for females (*range* = 18 to 32 years old).

Self-reported levels of previous familiarity between participants were rated by each individual regarding the other two in the triad using a Likert scale ranging from 1 to 7 (1 = “don’t know each other at all”; 7 = “know each other very well”). Overall, familiarity between individuals in the study was low but was significantly higher in the synchronized drumming condition (*M* = 2.19, *SD* = 1.03) than in the asynchronous drumming condition (*M* = 1.61, *SD* = 0.70): *t*(147) = 3.9, *p* = 0.0001, 95% *CI* = 0.28–0.86. Reported levels of musical knowledge were based on averaging the responses to the following three items: “I consider myself someone who has knowledge in music”; “I have been playing music from a young age”; and “I can read notes”. These three items were rated on a Likert scale ranging from 1 to 5 (1 = “not at all”; 5 = “very much so”) and had a mean of 1.90 (*SD* = 0.93). Since expert musicians were excluded from the study, the participants’ musical knowledge was relatively low. There were no significant differences between the groups in the synchronized condition and the groups in the asynchronized condition in terms of musical knowledge (*M* = 2.51, *SD* = 1.12; *M* = 2.24, *SD* = 1.07, for the synchronized and asynchronized conditions, respectively): *t(*148) = 1.53, *p* = 0.12, 95% *CI* = −0.07–0.63. Familiarity and musical experience were controlled for in the main model of the study.

### Procedure

The study was approved by the Department of Psychology’s IRB ethical committee at Bar-Ilan University and was conducted strictly according to the ethical guidelines. Prior to their arrival, participants were contacted by the study’s coordinator via email and were reminded to arrive to the study well hydrated and to avoid caffeinated drinks and nicotine for at least two hours prior to arrival. Upon arrival to the lab, the research assistant (RA) in charge of the study welcomed the participants and explained that in the following hour, they would be connected to electrodes collecting physiological data while performing several drumming tasks. The RA explained that data acquisition was not invasive or dangerous and would not hurt. In addition, he or she explained that the entire procedure would be videotaped and that the videos would be used only for data analysis by the research team and would not shared with third parties. After participants provided informed consent, they were individually connected to MindWare Mobile Recorders (MindWare Technology, Gahanna, OH) for physiological monitoring. The RA asked participants to avoid touching the electrodes or the recorder unit throughout the study. The participants were seated around a MIDI electronic drum set (see full details of the setup are given in the *Drumming Paradigm* section in the supplementary materials) throughout the experiment, and a taped loop in electrode lead cables was used to further limit the movement of artifacts. Participants were asked to limit the motion in their nondominant arm and to strictly drum using their dominant hand. Participants were also instructed not to talk to each other during the experiment (see further details on the practice leading to the group drumming task in the supplementary materials).

For half of the groups, the given tempo was unpredictable; thus, group members would be unable to synchronize perfectly with the beat, and therefore, the resulting group drumming was asynchronous. For the other half, the tempo was predictable and allowed group members to drum synchronously with each other. Each rhythmic pattern introduced in the practice round was 45 seconds in duration, with a 15-second pause between them. There were no differences in practice time between conditions or between groups. After the task, participants completed questionnaires assessing the experience of synchronization between them during the task, their affect and the level of group cohesion.

Physiological observation of all group members through ECGs was conducted simultaneously during the four separate concurrent phases. The first baseline phase lasted five minutes, in which participants were asked to sit down, relax and do nothing. The drumming task phase lasted 4 minutes. The second baseline phase (which was identical to the initial baseline phase) followed the drumming task. Finally, participants took part in a 4-minute drumming improvisation session in which they were instructed to freely play together on the drums. Participants were seated throughout the entire experiment. Throughout the procedure, all interactions among participants were videotaped from three angles (at 25 frames per second via video cameras temporally synchronized with each other and with the physiological recordings) in order to capture the faces and bodies of all group members.

## Measures

### Surveys

#### Demographic information and musical knowledge

A few days prior to each participant’s lab visit, a link to an online survey powered by Qualtrics, an online survey platform, was sent to the participant. In their responses to the survey, the participants reported their age, gender, and musical knowledge. Upon arrival to the lab, the participants were asked to report the last time they ate, drank, and consumed tobacco and if they had been diagnosed with cardiac disease or regularly took any medication. In addition, they were asked to indicate the degree to which they knew the other two group members (i.e., “prior familiarity”).

#### Group cohesion

We measured group cohesion with the following 4 items using a 1 to 6 Likert scale: “If possible, I would be happy to participate in another group experiment with the members of my current work group”; “My group worked together as a team”; “We were cooperative with each other”; and “We knew that we could rely on one another” and “We were supportive”. The relevant items were adopted from a well-validated questionnaire assessing cohesion^87^. The answers to all items were averaged to reach a group cohesion score for each individual. The scale reliability was high (Cronbach’s alpha = 0.92; McDonald’s omega = 0.92).

#### Perceived synchrony

To validate the study’s manipulation, we asked the participants to respond to two items following the drumming task – “I felt that we were playing in synchrony” and “I felt that we were playing in harmony” – using a 5-point Likert scale ranging from “not at all” to “very much”. There was a positive and significant correlation between the responses to both items: *r* = 0.798, *p* = 0.0001

#### Affect

Positive and negative affective states were measured using the PANAS^88^. This widely used scale measures positive and negative affect with 20 items: half are positive (e.g., “excited”) and half are negative (e.g., “hostile”). For each item, participants reported how they currently felt on a 1–5 Likert scale. The answers to all items were averaged to reach individual scores for positive affect and negative affect. The scales’ reliabilities were relatively high both for positive affect (Cronbach’s alpha = 0.83, 0.89, 0.91; McDonald’s omega = 0.84, 0.89, 0.91) and negative affect (Cronbach’s alpha = 0.83, 0.85, 0.87; McDonald’s omega = 0.83, 0.86, 0.87) for the first, second and third administrations of the survey, respectively.

### Physiological measures: IBIs of the Heart

#### Collection

An ECG was obtained for each group member using a modified lead II configuration. The impedance cardiogram was obtained using the standard tetrapolar electrode system, the procedures of which are described elsewhere^89^. The electrodes were connected to a MindWare Mobile Recorder unit for each individual and synchronously transmitted wirelessly to a laptop computer in the control room adjacent to the lab room. The sampling rate was 500 Hz.

#### Pre-processing

Each participant’s ECG signal was analyzed in MindWare Technology’s HRV application software, version 3.1.4. Visual inspection and manual editing of the data were completed by trained graduate students to ensure the proper removal of artifacts and ectopic beats^90^. The ECG signal was amplified by a gain of 1000 and filtered with a hamming windowing function. Due to measurement artifacts, we were unable to use ECG data for four participants. The IBI series was later spline interpolated using MATLAB 2019a (The MathWorks, Inc.) for every 500 milliseconds to obtain an equal interval time series. IBI represents the time in milliseconds between two heartbeats. Thus, as heart rate increases, IBI decreases. IBI is considered to be regulated by both sympathetic and parasympathetic branches of the ANS.

#### Assessing group-level physiological synchronization in IBI

The continuous IBI time series for each group member was later inputted to MATLAB, and a time-domain time-series analysis^91–93^ of the cross-correlation function (CCF) was employed. We defined a temporal lag of 3 seconds [data point lag = −6 to +6^58,59^] for the CCF to find the maximum dyadic correlations of IBI data between all pairs of group members. The results of the CCF analyses enabled us to assess the maximal degree of synchronicity between each dyad’s time series. Thus, the IBI cross-correlation between each of the three dyads in the group was captured. In Figures S1 and S2, we present detailed descriptive statistics regarding the dyad-level correlations computed from the CCF analyses. A group synchronization score was calculated as the mean of each group’s three dyadic maximum correlations. CCF analyses have been extensively utilized in research to examine the bivariate coupling strength in continuous data, specifically for IBI and heart rate^36,70^.

### Drumming paradigm and analysis

A full description of the drumming setup is described in the supplementary materials.

#### Analysis

The drumming data in MIDI format were analyzed via Ableton Max for Live, a platform for visual programming (Max/MSP). We measured the time that elapsed between each hit and then compared it across the three participants to check if all group members were hitting their drum pad together within a fixed threshold of 30 milliseconds. The hit analysis produced a binary state: hitting together versus not hitting together. Only times in which all three members hit their drums together within this window were counted to produce a performance group measure of coordination in drumming.

## Supplementary information

Supplementary Materials.

## Data Availability

The data that support the findings of this study are available from the corresponding author (I.G.), but restrictions apply to the availability of these data, which were used under license for the current study and are therefore not publicly available. Data are, however, available from the authors upon reasonable request and with permission of the corresponding author.
