# Supplementary material for: Physiological and Behavioral Synchrony Predict Group Cohesion and Performance
Source: Sci Rep. 2020 May 21;10:8484. doi: 10.1038/s41598-020-65670-1 (PMC7242382; doi:10.1038/s41598-020-65670-1)
Supplement: Supplementary file 1 — Supplementary Materials. [file 41598_2020_65670_MOESM1_ESM.pdf]

# Physiological and Behavioral Synchrony Predict Group Cohesion and Performance

Ilanit Gordon[1,2]*, Avi Gilboa[3], Shai Cohen[3], Nir Milstein[1], Nir Haimovich[1], Shay Pinhasi[5], Shahar Siegman[6]

[1]Department of Psychology, Bar-Ilan University, Ramat-Gan, Israel

[2]The Gonda Brain Research Center, Bar Ilan University, Ramat-Gan, Israel

[3]The Music Department, Bar Ilan University, Ramat-Gan, Israel

[4]The Psychology Department, Rupin College, Emeq-Hefer, Israel

[5]The Department of Computer Science, Bar Ilan University, Israel

**Corresponding author**: Ilanit Gordon, Ph.D., Department of Psychology and The Gonda Brain Research Center, Bar-Ilan University, Ramat-Gan, Israel. Tel: +972-3531- 7520, Email: ilush.gordon@gmail.com; Ilanit.gordon@biu.ac.il

**Keywords:** Physiological Synchrony, Group Cohesion, Group Performance, IBI, Drumming, Coordination

*Supplementary Materials*

*Practicing the Drumming Task.* Each participant was given an opportunity to get acquainted to drumming on their own and was asked to drum along with a metronome steady beat that was played stereophonically via two speakers while seated across from the drum set for 30 seconds.

Before the individual practice drumming task, we collected a 5-minute baseline recording of physiological measures in which participants were asked to simply sit together and relax, and not move or talk. Then, the group began practicing the joint group drumming task. Participants were told that just like in the individual drumming practice, they will each sit in front of their individual drum and drum together with 4 consecutive rhythm patterns that they will hear through the speakers. They were instructed to only beat on their designated drum. This practice phase was conducted once for every group and then we asked them if they felt they could perform the task. The group task began only when all group members indicated they were ready.

*Drumming Setup.* Participants played a Roland V-drum, a MIDI electronic drum set controller. To meet the requirements of the study, we modified it by removing the bass and high-hat pedals, leaving only the snare and two tam-tam drums. We employed the MIDI protocol for digital data collection; that is, hitting a drum pad comprised of three separate pads, generates its time stamp, note number, and velocity. The data were recorded using Ableton Live10—a software music sequencer and digital audio workstation. The rhythmic patterns that group members heard and attempted to match

with their drumming were played through the Ableton software which emulates the Roland drum machine sound and amplified through two speakers (a Samson Auro X12D powered active speaker and Gemini ES-08P). The four rhythmic patterns in the predictable condition consisted of incrementally increased complexity: (1) snare drum hit on every beat; (2) bass drum on beats 1 and 3 and snare drum on beats 2 and 4, alternating between accent and non-accent beat, creating a 4/4 time signature feel; (3) more complex rhythm including syncopation pattern; and (4) full complex drum groove that includes: bass and snare drums, clave and maracas. In the non-predictable experiment, the same 4 rhythmic patterns were played, but with radical changes in the tempo track.

*Supplementary Results*

*Assessing changes in affect from before and after the drumming task* - A Repeated Measures ANOVA was conducted with the task condition as a between-subject factor, and changes in positive and negative emotions from baseline to the post drumming task as the within-subject factors. Results showed no main effect for time in predicting changes in emotionality, $F(1,140) = .12$, $p = .729$, *partial* $eta^2 = .001$. Interaction effects revealed a significant interaction of condition*time on changes in affect, $F(2,139) = 4.5$, $p = .013$, *partial* $eta^2$=. 061, which was further tested using pairwise comparisons adjusted by a Bonferroni correction for multiple comparisons. Results showed that for only negative emotions, there were significant decreases in both task conditions. (*Mean Difference* = .088, $p = .029$, *95% CI* =. 009 - .168 and *Mean Difference* = .225, $p = .0001$, *95% CI* = .138 - .312 for the synchronized and asynchronized conditions, respectively). These results indicate that the asynchronous task condition was not

associated with an increase in negative emotionality or a decrease in positive emotionality that may account for enhanced cohesion.

*Supplementary Discussion*

Why were there no differences in affect between the conditions in the drumming task? A recent meta-analysis[1] showed that despite the fact that synchrony versus no synchrony had a significant and small effect size for increased positive affect, the contrast of synchrony versus asynchrony did not yield this impact [1] (pp. 18). Thus, not all research on synchrony's effects relate it to positive changes in mood. Moreover, since the aim of our drumming task was to test synchrony (and not mood), we did not want to elicit more positive or less negative moods in the synchronous condition compared to the asynchronous condition. Therefore, we made sure that the asynchronous task was not frustrating or difficult compared to the synchronous one for participants. We further made sure there would be changes in tempo complexities for all groups to maintain a similar degree of participant interest and alertness across all conditions. We kept each tempo relatively short, so not to be tiring or very boring. Finally, we should note here that research has shown that "positive emotions need not be generated for synchrony to foster cooperation" [2].

*Supplementary References*


1. Mogan, R., Fischer, R., & Bulbulia, J. A. (2017). To be in synchrony or not? A meta-analysis of synchrony's effects on behavior, perception, cognition and affect. *Journal of Experimental Social Psychology*, *72*, 13-20.
2. Wiltermuth, S. S., & Heath, C. (2009). Synchrony and cooperation. *Psychological science*, *20*(1), 1-5.

*Supplementary Tables*

*Table S1*

Group level correlation of the main variables in the study

Correlation Matrix

| | | Task condition | Physiological synchrony | Coordinated performance |
|---|---|---|---|---|
| Task condition | Pearson's r | — | | |
| | p-value | — | | |
| | 95% CI Upper | — | | |
| | 95% CI Lower | — | | |
| Physiological synchrony | Pearson's r | -0.025 | — | |
| | p-value | 0.566 | — | |
| | 95% CI Upper | 1.000 | — | |
| | 95% CI Lower | -0.266 | — | |
| Coordinated performance | Pearson's r | 0.241 * | 0.306 * | — |
| | p-value | 0.046 | 0.018 | — |
| | 95% CI Upper | 1.000 | 1.000 | — |
| | 95% CI Lower | 0.006 | 0.069 | — |

*Note.* $H_a$ is positive correlation

*Note.* * $p < .05$, ** $p < .01$, *** $p < .001$, one-tailed

*Table S2*

Individual level correlations of the main variables in the study

Correlation Matrix

| | | Drumming condition | Physiological synchrony | Cohesion | Perceived synchrony | Positive affect | Negative affect |
|---|---|---|---|---|---|---|---|
| Drumming condition | Pearson's r | — | | | | | |
| | p-value | — | | | | | |
| | 95% CI Upper | — | | | | | |
| | 95% CI Lower | — | | | | | |
| Physiological synchrony | Pearson's r | 0.025 | — | | | | |
| | p-value | 0.384 | — | | | | |
| | 95% CI Upper | 1.000 | — | | | | |
| | 95% CI Lower | -0.114 | — | | | | |
| Cohesion | Pearson's r | 0.196 ** | 0.236 ** | — | | | |
| | p-value | 0.008 | 0.003 | — | | | |
| | 95% CI Upper | 1.000 | 1.000 | — | | | |
| | 95% CI Lower | 0.063 | 0.099 | — | | | |
| Perceived synchrony | Pearson's r | 0.178 * | 0.006 | 0.275 *** | — | | |
| | p-value | 0.015 | 0.474 | < .001 | — | | |
| | 95% CI Upper | 1.000 | 1.000 | 1.000 | — | | |
| | 95% CI Lower | 0.044 | -0.134 | 0.145 | — | | |
| Positive affect | Pearson's r | -0.087 | -0.063 | 0.068 | 0.288 *** | — | |
| | p-value | 0.855 | 0.772 | 0.205 | < .001 | — | |
| | 95% CI Upper | 1.000 | 1.000 | 1.000 | 1.000 | — | |
| | 95% CI Lower | -0.220 | -0.201 | -0.068 | 0.159 | — | |
| Negative affect | Pearson's r | 0.040 | -0.028 | 0.001 | 0.002 | 0.243 ** | — |
| | p-value | 0.315 | 0.628 | 0.494 | 0.488 | 0.001 | — |
| | 95% CI Upper | 1.000 | 1.000 | 1.000 | 1.000 | 1.000 | — |
| | 95% CI Lower | -0.096 | -0.167 | -0.134 | -0.133 | 0.112 | — |

*Note.* $H_a$ is positive correlation

*Note.* * p < .05, ** p < .01, *** p < .001, one-tailed

*Supplementary Figures*

Figure S1

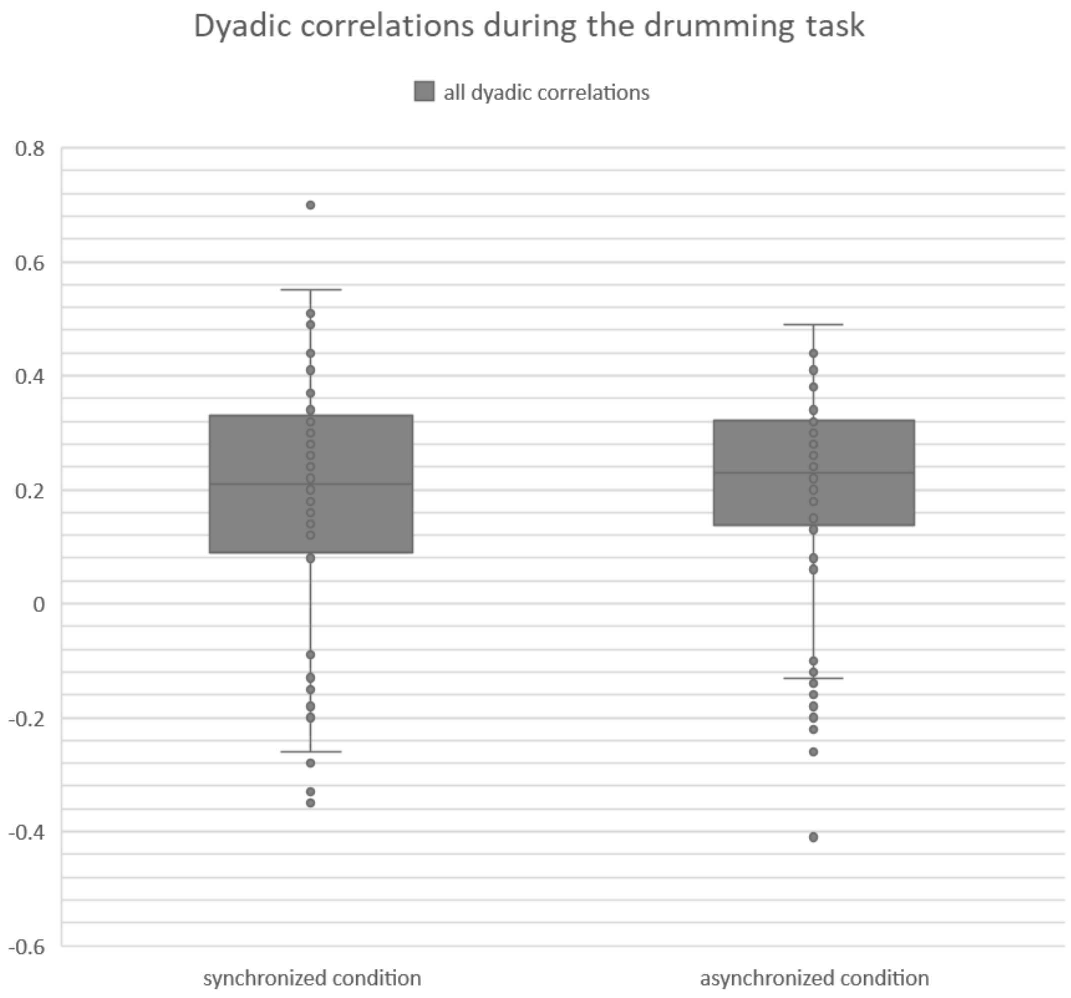


**Figure S1**. Box plot depicting the standardized correlation coefficient for each dyadic IBI correlation score in the synchronized and a-synchronized drumming task conditions. Each dot represents a single dyadic correlation calculated correlation for each group. The horizontal line in the blue box represents the median, the box represents the interquartile range (IQR) and the vertical lines represent Q1-1.5*IQR and Q3+1.5*IQR. As can be seen, dyadic level correlations was similar between the two task conditions.

Figure S2

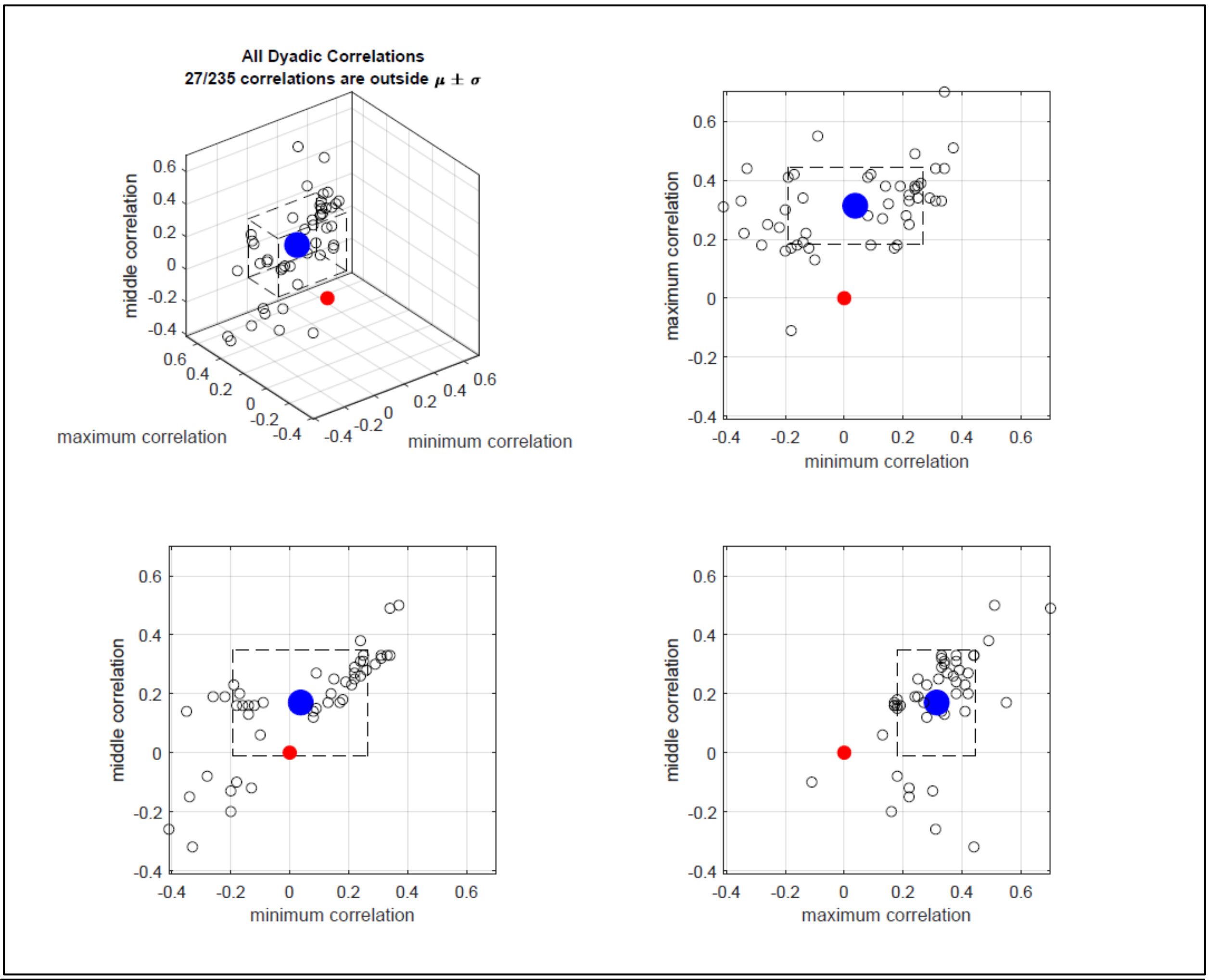


**Figure S2**. A 3-D scatter plot representing the minimum, maximum and medium level dyadic correlations comprising each group in the study. The red dot represents coordinates (0,0,0). The Blue dot represents the average dyadic correlation in each dimension. The dotted box represents 1 *SD* beyond the average. We sparse out the 3-D graph to three two-dimensional graphs that will make all dimensions easier to view and interpret.
